# Comparative Evaluation of Three Different Techniques of Radial Artery Cannulation: A Prospective Randomised Study

**DOI:** 10.7759/cureus.52326

**Published:** 2024-01-15

**Authors:** Deepak Singla, Mishu Mangla, Ankit Agarwal, Ranjeeta Kumari

**Affiliations:** 1 Department of Anaesthesiology, All India Institute of Medical Sciences, Rishikesh, IND; 2 Department of Obstetrics and Gynaecology, All India Institute of Medical Sciences, Hyderabad, IND; 3 Department of Community and Family Medicine, All India Institute of Medical Sciences, Rishikesh, IND

**Keywords:** complications, ultrasound machines, equipment, measurement, arterial pressure

## Abstract

Objective: We planned this study to compare three approaches to arterial cannulation, i.e., catheter over the needle, catheter over the guidewire, and ultrasound-guided cannulation, in terms of overall success rate, first pass success rate, time for cannulation and incidence of complications.

Methods: After obtaining informed written consent from the patient, they were randomized into three groups, based on chits in the box technique, to undergo radial artery cannulation as follows: group N (using catheter over needle technique), group W (using catheter over guidewire technique), group U (radial artery cannulations under ultrasound guidance). We calculated a sample size of 50 patients in each group based on the primary endpoint of the overall success rate. The data was analyzed using one-way ANOVA and post hoc Tukey's test.

Results: There was a non-statistically significant trend towards a higher overall success rate in groups W and U compared to group N (47 and 46, respectively, compared to 43, p-value 0.35). Similarly, no significant differences were observed concerning any of the characteristics of radial artery cannulation, except the first pass success rate, where the success rate was highest in group W (33, 70.21%), followed by group U (34, 68%) with a p-value of 0.04.

Conclusion: Though catheter over guidewire and ultrasound-based techniques offer advantages in terms of higher first-pass success rate, they do not significantly increase the overall success rate or reduce the total incidence of complications.

## Introduction

Arterial blood pressure is a fundamental cardiovascular vital sign, and arterial cannulation with continuous pressure transducer remains the accepted reference standard for arterial blood pressure monitoring, in critical situations. Invasive arterial blood pressure was found to correlate more with cerebral blood flow compared to the noninvasive method [1]. It provides crucial and timely information in intraoperative and critically ill patients, outweighing its risks. It is superior to indirect blood pressure monitoring in the early detection of hypotension, especially in critical care patients. The arterial pressure waveform also provides valuable information regarding fluid responsiveness by calculating pulse pressure variation. It also aids repeated arterial blood sampling and diagnostic and therapeutic procedures like vascular stenting and arterial embolization.

The most common site for arterial cannulation is the radial artery because of good collateral circulation, consistent anatomic accessibility, ease of cannulation, and low rate of complications [2]. Other sites include the ulnar, axillary, femoral, dorsalis pedis, posterior tibial, and superficial temporal artery. The most common techniques for arterial cannulation are catheter over needle and catheter over guidewire technique. One study showed that the guidewire technique is superior to the direct technique in male patients [3]. Arterial cannulation can also be done under ultrasound (USG) guidance. Studies have shown that ultrasound-guided arterial cannulation resulted in greater success rate than cannulation by palpation [4]. The choice of method depends on the operator's preference and availability of equipment. However, arterial cannulation is not without complications, which include distal ischemia, pseudoaneurysm, arteriovenous fistula, haemorrhage, arterial embolization, infection, and peripheral neuropathy [5].

Very few studies have evaluated the relative efficacy of these three techniques i.e., catheter over the needle, catheter over guidewire and ultrasound-guided cannulation. So, we planned this study to compare these three techniques in terms of overall success rate, first pass success rate, time for cannulation and incidence of complications. This study was previously presented in 12th ISA-Central Zone and 12th ISA Uttarakhand state on 28 and 29 October 2023.

## Materials and methods

We have planned to conduct the study on radial artery cannulation as there is extensive collateral circulation, easily accessible for cannulation, comfortable to patients and fewer complications. Before starting the study, institutional ethics approval was obtained (11/IEC/IM/NF/2017). Patients were selected based on the inclusion and exclusion criteria as described below. Adult patients aged 18 to 60 years undergoing elective surgery requiring continuous arterial pressure monitoring were considered for our study. Exclusion criteria were hemodynamically unstable patients (mean arterial pressure (MAP) > 50 mm of Hg or need for vasopressors to maintain MAP > 60 mm of Hg), skin infection or an abscess at the puncture site, negative modified Allen's test, recent arterial puncture, patients requiring emergency surgery.

After obtaining an informed written consent from the patient, they were randomized into three groups based on the chit in box technique [6] as: group N - underwent radial artery cannulation using a catheter over needle technique (n = 50); group W - underwent radial artery cannulation using a catheter over guidewire technique (n = 50), and group U - underwent radial artery cannulations under ultrasound guidance (n=50).

The modified Allen test [7] was done to confirm collateral circulation. The patients were asked to clench their fists tightly for one minute, pressure was applied over both radial and ulnar arteries simultaneously so as to occlude them. The patients were then asked to open their fingers rapidly. The rapidity with which initial pallor is replaced by standard color was assessed, typically returns by five to 15 seconds (a positive modified Allen's test). After the procedure was explained to the patient, the arm was kept in an arm board in 30-60 degrees of dorsiflexion. The volar aspect of the wrist was prepared by spirit or chlorhexidine solution and draped using sterile techniques.

In group N, the radial artery was palpated. Under local lidocaine infiltration, a radial artery was punctured at the site of maximum radial artery pulsation using 20G cannula at an angle of 30 to 60 degrees to the skin. The needle and cannula were advanced until blood return was noticed in the hub, and then the needle was introduced slightly. The needle and cannula were flattened to the skin, the needle slightly withdrawn, and the cannula was advanced into the artery. Correct positioning was confirmed by pulsatile blood flow when the needle was removed. 

In group W, the percutaneous puncture was made similarly, but with needle, catheter and guidewire assembly (Arrow RA-04120 radial artery catheterization set, 20G). We attached a syringe to the finder needle. Then, the needle was advanced with slight negative pressure until free return of blood was visualized in the syringe. Once the free flow of blood was obtained, the syringe was removed, and the guide wire was advanced into the artery. Then we removed the needle while holding the guide wire in place and advanced a catheter over the wire into the artery. Correct positioning was confirmed by pulsatile blood flow when the needle was removed.

**Figure 1:**
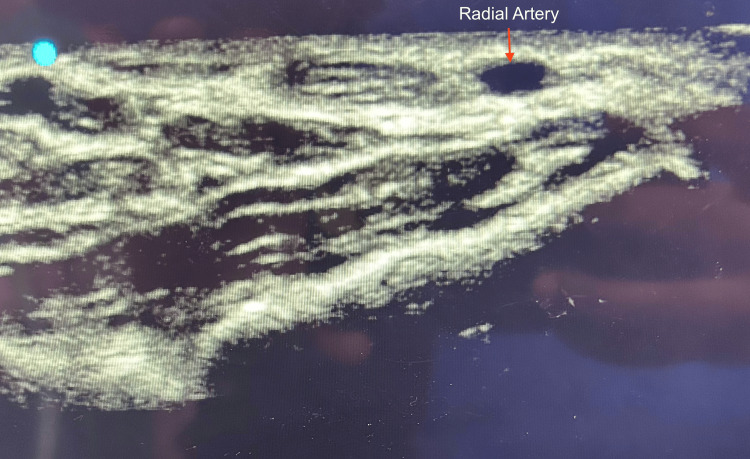
Ultrasound view of radial artery (transverse section)

**Figure 2:**
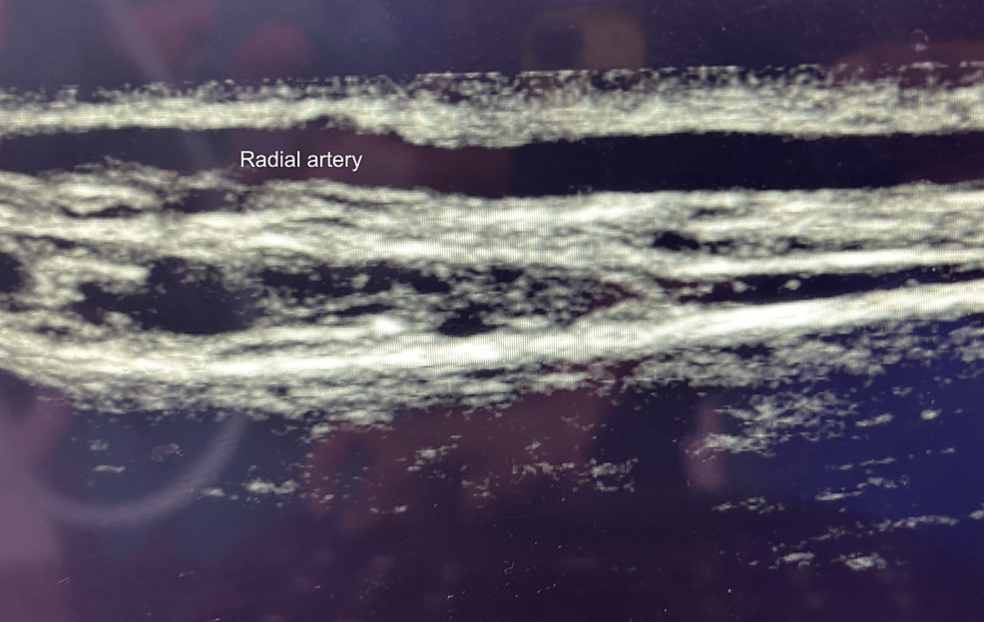
Ultrasound view of radial artery (longitudinal section)

In group U, radial artery cannulation was done using a linear ultrasound probe. After preparation of the wrist to be punctured, we placed the linear probe (8-12 MHz, Sonosite Edge II) on the skin in cross-sectional area (transverse section) to visualize the radial artery (Figures 1, 2).

**Figure 3:**
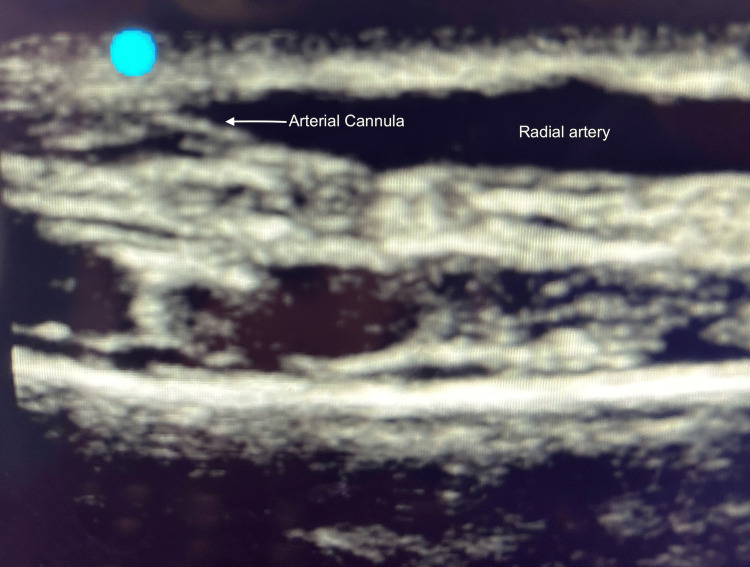
Arterial cannula in radial artery

**Figure 4:**
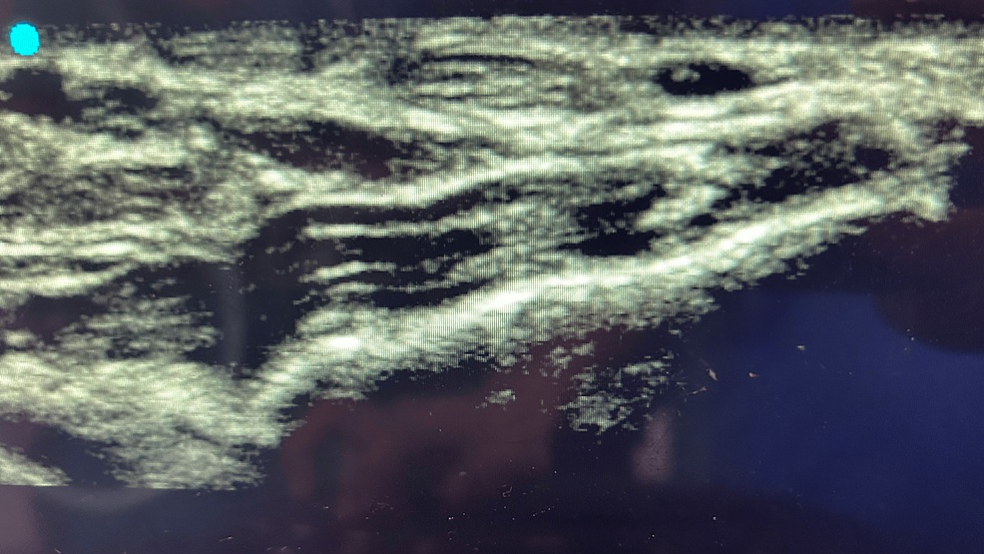
Radial artery with arterial cannula (transverse view)

An arterial cannula (20G) was then advanced under ultrasound guidance until the artery was punctured (confirmed by the appearance of bright red blood in the hub of the needle) (Figures 3, 4).

Ultrasound was removed, and the rest of the process was completed using the same technique as in group N.

The primary objective for this study was the overall rate of successful cannulation in all three groups. Secondary objectives were the average number of insertions required for successful cannulation, first-pass success rate (the total number of insertions done in the first attempt), time taken for successful arterial cannulation and failure rate was analyzed and compared. An attempt was considered if a skin puncture was made over the radial artery, and failure was an inability to cannulate the radial artery within five minutes, or three or more attempts were required for cannulation. In groups N and W, time was measured from when the anesthesiologist placed his figures over the wrist for digital palpation of the radial artery and in group U from the time of placement of the ultrasound probe over the wrist. The time of successful cannulation was noted from skin puncture till the appearance of a radial artery trace on the monitor (as done in similar previous studies) [8].

Complications like bleeding, hematoma formation, infection, distal discoloration/skin necrosis, digital gangrene, pseudoaneurysm formation and median nerve injury were noted. For this the patients were monitored by careful observation and daily visits for three days after the procedure. Bleeding was defined as any amount of visible bleeding at the site of arterial puncture. Hematoma formation was defined as appearance of visible swelling either during or a few minutes after the arterial puncture is attempted. Infection was the presence of signs of inflammation (redness, tenderness), and/or discharge at the site of arterial puncture. Development of bluish or blackish discolouration of skin was classified as distal discoloration/skin necrosis and if it involved entire fungus or was associated with swelling, necrotic changes, it was classified as digital gangrene. Pseudoaneurysm was defined as a swollen radial artery as visible on ultrasound after 48 hours. And median nerve injury included and altered sensation, tingling, numbness in the area supplied by median nerve distal to the site of arterial puncture not associated with any change in skin colour.

Based on similar studies done in the past [8] the overall success rate among patients undergoing radial artery cannulation with catheter over needle technique and catheter over guide wire technique was found to be 96% (48 out of 50 patients) vs 76% (38 out of 50 patients). So, using a confidence level of 95% and power of study of 80% the minimum number of patients required was found to be 44 patients. Similarly, in previous studies [9] overall success rate with catheter over needle technique and under ultrasound guidance was found to be 89.4% (118 out of 132 patients) vs 64.84% (83 out of 128 patients). At a confidence level of 95% and power of study of 80%, the minimum number of patients required was 42. So, we included 50 patients in each group to have better accuracy.

Continuous variables were analyzed using one-way ANOVA to compare them across the three groups. Post hoc Tukey's test was applied to find out which of the three pairs exhibited statistically significant differences. The Chi-square test was applied to test for the hypothesis of no difference between the three groups with respect to categorical variables. A p-value of <0.05 was considered significant and rejected the null hypothesis of no difference across the three groups.

## Results

**Figure 5:**
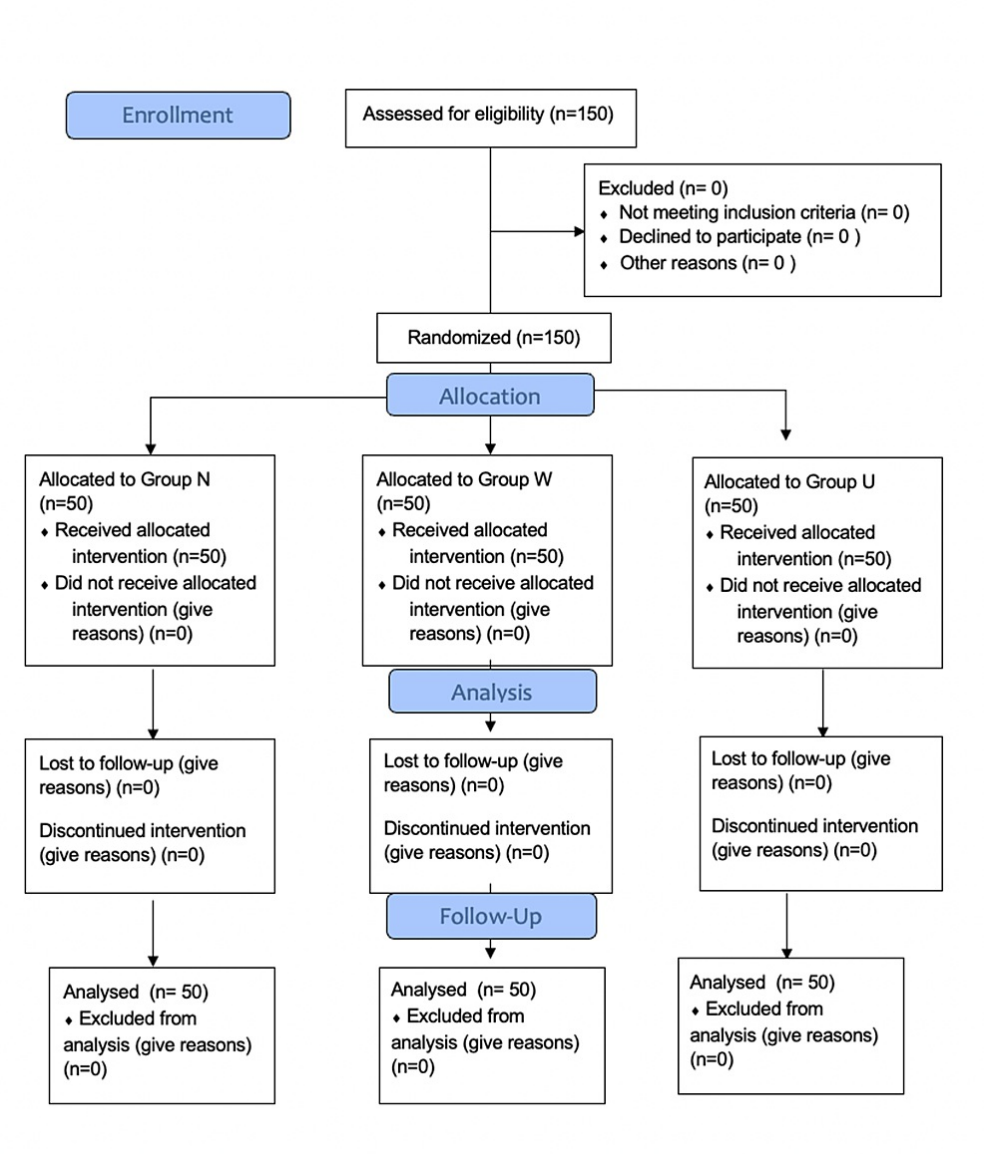
CONSORT 2010 Flow Diagram

In total 150 patients were considered for this study. No patients were excluded, and all the patients finished the study (Figure 5).

Baseline comparison of various variables (Table 1) showed that the three groups were comparable with respect to all the parameters (p>0.05).

**Table 1:** Comparative demographic profile of patients in three groups

Demographic profile	Group N	Group W	Group U	p value
Age (Mean, SD)	43.34, 17.59	40.58, 15.74	39.9, 17.63	0.56
Gender (No., %)				
Male	20, 40%	25, 50%	24, 48%	0.56
Female	30, 60%	25, 50%	26, 52%		
BMI (Mean, SD)	24.68, 3.298	24.52, 3.621	24.54, 3.887	0.97
Heart rate (Mean, SD)	74.52, 12.25	75.52, 14.31	74.4, 14.34	0.90
MAP (Mean, SD)	77.36, 13.69	77.42, 11.59	75.8, 11.94	0.76
No.– number, % - percentage, SD-standard deviation				

We compared the three groups in terms of various parameters (mentioned above) in Table 2. Though the overall success rate was higher in groups W and U as compared to group N (47 and 46, respectively compared to 43), the difference was not statistically significant (p-value 0.35). We did find a statistically significant difference in the first pass success rate, where the success rate was highest in group U (34, 73.9%), followed by group W (33, 70.21%) and lowest in group N (24, 55.81%) with a p-value of 0.04. No significant differences were observed between the groups with respect to any other characteristics of radial artery cannulation.

**Table 2:** Characteristics of radial artery cannulation of patients in different groups

Characteristics of Radial Artery cannulation	Group N	Group W	Group U	Test statistic, p value
Cannulation successful (No., %)				
Yes	43, 86	47, 94	46, 92	0.04, 0.35
No	7, 14	3, 6	4, 8		
Number of attempts No, %				
1	24, 55.81	36, 70.21	34, 73.91	4.76, 0.31
2	11, 25.28	10, 21.28	6, 13.04	
≥3	8, 18.61	4, 8.51	6, 12.77	
First pass success rate (No., %)				
Yes	24, 55.81	33, 70.21	34, 73.9	6.26, 0.04
No	19, 44.18	14, 29.79	12, 26.08	
Time to cannulate (Mean, SD)	88.58, 29.48	84.04, 25.53	84.93, 32.50	0.29, 0.74
Complications (No., %)				
Yes	11, 22	5,10	8, 16	2.67, 0.26
No	39, 78	45, 90	42, 84		
No.– number, % - percentage, SD-standard deviation					

In group comparison (Table 3), first pass success rate was significantly higher in group W compared to group N and in group U compared to group N. However no significant difference was observed between group W and U. 

**Table 3:** Group comparison for first-pass success rate

First pass success rate	Test static	P value	
N vs W	4.8	0.0286	
N vs U	7.9	0.005	
W vs U	0.39	0.528	

Through higher number of complications were observed in group N and least in group W the difference was not statistically significant. Eleven patients in group N (eight hemorrhage, three hematoma), five patients in group W (three hemorrhage, two hematoma), eight patients in group U (six hemorrhage, two hematoma), developed complications. No other complications were noted in any patient in our study.

## Discussion

Our study aimed to compare three different techniques of radial artery cannulation in terms of ease of catheterization, overall success rate, first pass success rate, number of attempts and incidence of complications. The overall success rate trended towards being higher in groups W (94%) and U (92%), though the difference was not found to be statistically significant. Our study showed that group U, using ultrasound, had the highest first pass success rate, followed by group W, i.e., catheter over the guide wire, and group N, using the conventional technique of inserting catheter over the needle. The incidence of complications was least in group W and maximum in group N, although it did not reach statistical significance.

In our study, though the overall cannulation success rate was higher in group U (92%) and W (94%), as compared to group N (86%), but it was not statistically significant (p=0.35). Few other studies also compared the overall success rate of arterial cannulation using different methods and reported similar outcomes. Ueda et al. [10] conducted a study in 104 patients, who underwent radial artery cannulation by either Doppler-guided or USG-guided method. The USG group had a higher percentage of successful cannulation within 10 minutes (65%) in comparison to the Doppler group (46%). Manger et al. [3] observed an overall success rate with guidewire at 82% compared with that of the direct method of 62% (p=0.02). However, a study conducted on experienced anesthesiologists [11] did not find any difference between the overall success rate when ultrasound was used for arterial cannulation as compared to direct palpation.

Our study found that the use of ultrasound (group U) and guidewire (group W) resulted in a higher first-pass success rate than group N. It increased from 55.81% in group N to 73.9% in group U and to 70.21% in group W. In a study conducted among experienced anesthesiologists (attending Canadian cardiac anaesthesiologists with prior experience in US-guided radial artery cannulation) [11], the use of ultrasound resulted in a higher first-pass success rate, though the difference was not found to be statistically significant. In another study [12], the use of ultrasound resulted in a higher success rate and lower time of insertion as compared to the palpation technique. Similarly, Levin et al. [13] found in 69 adult patients requiring intraoperative monitoring, an improvement in first-pass success rate from 34% to 62% with ultrasound. 

Schwemmer et al. [14] found in 30 patients that the first-pass success rate increased from 20% to 67%. Similar findings were noted in a study by Shiver et al., [15] where radial artery cannulation was performed in 60 adult patients in the emergency department by either palpation or USG-guided method. They showed in critically ill patients, an improvement in first-pass success rate from 50% to 87% with the use of ultrasound. The first-pass success rate with USG in our study was lower than that of a study by Roberts et al. [16] (80%), where a single interventional cardiologist with very minimal previous experience of USG, performed radial artery cannulation in 50 patients under USG guidance. The conflict in results can be attributed to different patient groups i.e., emergency/critical patients used in different studies. We considered only elective patients for our study.

We further found that the time to cannulate was not significantly different among the three groups. A study was done by Levin et al. [13] where they compared the USG-guided method with the palpation method in 69 adult patients and found that the USG group required significantly more time to cannulate (26.1s) as compared to the palpation group (17.3s). Even the mean time for successful cannulation in the first attempt was more in the USG group. Yeldrim et al. [8] found that radial artery cannulation was successful in 48 patients in the guidewire insertion technique but only 38 in the direct needle catheter method. Mean elapse time was 22+/-4.6 minutes in the direct method and 7+/-4.2 minutes in the guidewire method. The mean number of attempts required for cannulation was 4.5 in the direct method and 2.1 in the guidewire method. The authors recommended using guidewire-assisted radial artery cannulation rather than a direct technique. 

Our study found that the complication rate was high, 22% in group N, followed by group U (16%) and least in group W (10%). A review by Scheer et al. [17] noted that the most common complications of radial artery cannulation be temporary artery occlusion (19.7%) and hematoma formation, local site infection (0.7%), haemorrhage and infrequently a pseudoaneurysm formation. However, we did not encounter cases of distal ischemia, local site infection or thrombosis. Seto et al. [18] compared 1004 patients who underwent femoral arterial cannulation under either fluoroscopy or USG guidance and found that the USG group had a significantly lower incidence of complications [1.4% (USG group) vs 3.4% (fluoroscopy group), p=0.04]. However, most studies determining the incidence of complications following radial artery cannulation have found the incidence to be quite low. A study regarding patient and risk factors for radial artery catheter-related complications found the incidence to be very low (3.4 per 10,000 patients) [19]. The reason for a higher incidence of complications in our study can be that we noted even minor bleeding as a complication for radial artery cannulation. 

Our study had some limitations. Firstly, ours being a single-center study, the sample size was kept small (150 patients). A multicenter study with a greater sample size can probably better establish the superiority or inferiority of one method over another. Secondly, we excluded the patients in shock or those requiring vasopressor support. So, remains to be seen how these three techniques compare in such patients. Lastly, if combining guidewire and ultrasound could further improve the success rate remains to be seen. Also, we only considered elective patients for our study. So, further studies are required to analyze the utility of ultrasound or guidewire-based radial artery cannulation in particular patient groups like emergency patients, morbidly obese patients, or patients with severe hypotension or on vasopressors.

## Conclusions

In conclusion, catheter over the guidewire and USG-guided cannulation were found to be superior to the direct palpation technique for radial artery cannulation, having significantly higher first-pass success rates of cannulation. No statistically significant differences were noted among the three groups, in terms of number of attempts for cannulation, time taken to successfully cannulate the radial artery and overall success rate. So, for the radial artery cannulation, both the guidewire-based and USG-guided methods were superior to the direct palpation method, and significantly improved the success rate in first attempt.
